# The clinical utility of autoantibodies in systemic sclerosis: a review with a focus on cohort differences and standardization

**DOI:** 10.3389/fimmu.2025.1691988

**Published:** 2025-10-24

**Authors:** Kazuhiro Komura

**Affiliations:** Department of Dermatology, Kanazawa Red Cross Hospital, Kanazawa, Japan

**Keywords:** systemic sclerosis, autoantibodies, ACA, ATA/Scl-70, RNA polymerase III, nucleolar antibodies, PM-Scl, Ku

## Abstract

**Background:**

Systemic sclerosis (SSc) is clinically heterogeneous. Disease-specific autoantibodies—anticentromere (ACA), anti–topoisomerase I (ATA/Scl-70), and anti–RNA polymerase III (RNAP III)—are central to classification and organ-risk prediction. Beyond prognosis, SSc-specific autoantibodies can support diagnosis as part of a composite assessment with nailfold capillaroscopy and clinical features; their contribution is reflected in the 2013 ACR/EULAR classification criteria and can be informative in very-early or sine presentations. More broadly, these immune signatures underpin routine SSc care and underscore the immunological impacts that shape disease expression.

**Methods:**

Narrative review (2000–August 2025) prioritizing studies in Japanese and Western cohorts, with emphasis on assay performance and cohort comparability. We appraise line immunoassay (LIA) performance vis-à-vis immunoprecipitation (IP), and integrate ICAP-compliant ANA interpretation.

**Results:**

ACA aligns with lower ILD risk but higher PAH and digital vasculopathy; ATA predicts ILD onset/progression; RNAP III marks rapid skin thickening, SRC risk, and temporally clustered malignancy; U1 RNP tracks overlap/MCTD-like features and PAH; U3 RNP indicates diffuse disease with vasculopathy; Th/To varies by center; PM-Scl and Ku flag overlap ILD/myositis. A clinical-first standardized workflow—ANA (ICAP) + core ELISAs (ACA, ATA, RNAP III, U1 RNP) followed by ANA-pattern–guided LIA/IP confirmation—supports both care and cross-cohort comparability.

**Conclusions:**

Autoantibodies form a practical foundation for SSc care across regions. Standardizing the reflex layer (LIA/IP) while leveraging established ANA and core ELISAs can reduce measurement-driven cohort differences and improve global synthesis of SSc evidence.

## Introduction

Autoantibodies are detected in most patients with SSc and map onto distinct clinical courses and organ risks (1). Integrating autoantibody status with cutaneous subset and disease duration improves prediction of morbidity and mortality. In addition to prognostic use, SSc-specific autoantibodies can support diagnostic classification—particularly when skin thickening is minimal (very-early SSc or sine scleroderma) (2) —complementing nailfold capillaroscopy as part of a composite assessment (3). More broadly, these immune markers form a practical foundation for SSc care and confirm the immunological impacts that shape disease expression. Multiplex line-blot assays, although practical for broad screening, show antigen-dependent variability necessitating orthogonal confirmation in key scenarios. Importantly, their distribution varies by ethnicity and geographic region. While many reports from Western cohorts are well characterized, Asian data—though more fragmented—are increasingly available. Recognizing both intra-Asian diversity and overarching trends is essential for accurate international comparison and for designing standardized, regionally sensitive testing strategies.

## Methods

This review was conducted as a narrative synthesis rather than a systematic review. We searched PubMed and Embase (January 2000 – August 2025) using a combination of terms including systemic sclerosis, autoantibodies, cohort, ACA, ATA, RNA polymerase III, nucleolar antibodies, U1 RNP, U3 RNP, U11/U12 RNP, ethnic differences, *and* interstitial lung disease.

Studies were included if they: (i) involved human SSc cohorts with serological data; (ii) reported clinical or prognostic associations of specific autoantibodies; and (iii) specified geographic or ethnic origin when relevant. We prioritized larger cohort studies, multi-center reports, and studies using validated assays (ELISA, LIA, IP, or ICAP-based ANA). Case reports and abstracts without peer-reviewed data were excluded. Additional references were identified by hand-searching the bibliographies of key articles.

## Results and discussion

### Autoantibodies and clinical phenotypes

#### Anticentromere

Clinical phenotype — Typically associated with limited cutaneous SSc (lcSSc). Vascular-dominant features include long-standing Raynaud phenomenon, telangiectasia, digital ischemia/ulcers, pitting scars, and calcinosis cutis (4). GI involvement is common (esophageal dysmotility, reflux). Tendon friction rubs are uncommon. Myopathy is rare, and inflammatory arthritis is usually mild if present.

Internal organ risk — Lower risk of fibrotic interstitial lung disease (ILD) compared with ATA, but clearly increased risk of pulmonary arterial hypertension (PAH), especially after >5–10 years of disease (5). Cardiac involvement most often reflects PAH/right heart strain rather than primary myocarditis. Renal crisis is uncommon. Malignancy risk is not specifically increased versus background in most cohorts.

Clinical course — Skin fibrosis tends to be mild and slowly progressive or plateauing. Morbidity is driven by vasculopathy (recurrent digital ischemia/ulcers) and PAH developing in the later course. Survival is favorable when PAH is screened and treated early; digital ulcer burden and recurrent ischemic events predict disability. Annual PAH screening is recommended, with baseline HRCT/PFT to document lung status even when ILD risk is low.

### Anti–topoisomerase I (ATA/Scl-70)

Clinical phenotype — Enriched in diffuse cutaneous SSc (dcSSc), but also present in a subset of lcSSc who nonetheless share high lung risk (6). Raynaud phenomenon is common but often of shorter duration before the onset of skin thickening (4). Digital ulcers were significantly associated with the presence of ATA (7). Flexor tendon friction rubs and rapid skin progression in the first 1–3 years are typical in dcSSc. Musculoskeletal pain and inflammatory arthritis may occur; calcinosis is less prominent than in ACA.

Internal organ risk — Strongly associated with ILD (predominantly NSIP on HRCT) with earlier onset, greater extent, and faster decline in FVC and DLCO during the initial years (8). Cardiac involvement includes myocardial inflammation, conduction abnormalities, and ventricular ectopy; PH can be secondary to parenchymal lung disease. Renal crisis risk is lower than in RNAP III but not negligible. GI involvement (esophageal hypomotility, reflux, small-bowel dysmotility) contributes to weight loss and malnutrition in advanced disease.

Clinical course — Skin thickening often peaks early (1–3 years) and then softens; lung disease progression is the major driver of disability and mortality. Early identification of progressive fibrosing ILD and prompt initiation of disease-modifying therapy (e.g., antifibrotics or immunomodulators per guideline) can alter the trajectory (9, 10). Close PFT monitoring (every 3–6 months initially) and HRCT reassessment are advisable.

### Anti–SS-A (Ro)

Although anti–SS-A (Ro) antibodies are not specific to systemic sclerosis, they are occasionally detected in patients with the disease, particularly in those with interstitial lung disease. Several cohort studies have shown that SS-A positivity correlates with a higher prevalence and severity of ILD and may also indicate overlap features with other connective-tissue diseases (11, 12). SS-A antibodies often coexist with other autoantibodies, and while their specificity for SSc is limited, they may reflect broader immune activation. In such cases, the clinical phenotype frequently combines the characteristics of both SS-A and the coexisting autoantibody, providing a composite picture that can influence disease presentation and management. Including SS-A testing may therefore provide additional practical information when evaluating SSc-ILD phenotypes.

### Anti–RNA polymerase III

Clinical phenotype — Often dcSSc or rapidly progressive skin thickening irrespective of cutaneous subset. Early edematous hands, new-onset tendon friction rubs, and abrupt rise in mRSS are common. Calcinosis and inflammatory arthritis can coexist. Raynaud phenomenon may be of short duration prior to skin involvement.

Internal organ risk — Markedly elevated risk of scleroderma renal crisis (SRC) (13). Hypertension may be absent initially; microangiopathic hemolytic anemia and rising creatinine can follow swiftly. Cancer clustering around disease onset (especially breast and hematologic) is reported in several cohorts (14). Lung involvement may be less extensive than in ATA, but not absent; PAH risk is not specifically high unless other risk factors coexist.

Clinical course — The first 1–2 years are critical. Home BP monitoring, avoidance of high-dose glucocorticoids, and immediate ACE-inhibitor therapy at SRC signal are lifesaving. When present, malignancy is often temporally close to SSc onset; individualized malignancy surveillance is reasonable early (15–17). Skin activity is front-loaded; long-term course depends on renal preservation and early event control.

### Fibrillarin (U3 RNP)

Clinical phenotype — Anti-fibrillarin (U3 RNP) is frequently associated with younger age at onset and dcSSc. Prominent vasculopathy (digital ulcers), extensive telangiectasia, and GI dysmotility (esophageal and small-bowel) are common (18). Myopathy and cardiac involvement (conduction abnormalities, myocarditis) are variably reported (19–21).

Internal organ risk — Both ILD and PAH are observed; PAH can occur even without extensive ILD (22). Right heart involvement may predominate in some patients. Ethnic variability exists, with stronger signals for aggressive disease in certain ancestries.

Clinical course — Earlier onset and diffuse skin involvement can confer a more aggressive first-phase course. Longitudinal monitoring should prioritize PAH screening and ILD evaluation, with early referral to expert centers for right-heart assessment when dyspnea or syncope appears.

### Th/To

Clinical phenotype — Typically lcSSc with nucleolar ANA pattern (23). Raynaud phenomenon, telangiectasia, and GI involvement are common; calcinosis may occur. Muscular and articular inflammatory features are uncommon compared with PM-Scl overlap.

Internal organ risk — ILD and/or PH occur at variable frequencies across centers; several series suggest a slower ILD progression and favorable long-term survival compared with ATA-ILD, but a non-trivial ILD/PH burden still warrants structured surveillance (3).

Clinical course — Skin disease is usually limited and indolent. Given inter-center variability, individualized ILD/PH screening strategies are appropriate; treatment responses are generally favorable when disease is detected early.

### NOR90 (hUBF)

Clinical phenotype — Rare. Reports suggest milder skin disease and fewer GI symptoms compared with other nucleolar antibodies, but data are inconsistent. Raynaud phenomenon is common; calcinosis is not a defining feature (24).

Internal organ risk — Associations with ILD or PAH are inconsistent; some series report low rates while others note clinically relevant lung involvement. No strong association with SRC or malignancy has been established.

Clinical course — Because of rarity and modest effect sizes, NOR90 positivity should be interpreted in the phenotype context. Routine surveillance (lung, PAH) as per SSc standard of care is advised.

### PM-Scl (PM/Scl-75/PM/Scl-100)

Clinical phenotype — Overlap phenotype with myositis is the hallmark. Proximal muscle weakness, elevated CK, and myopathic EMG are typical. Cutaneous disease is often limited or sine; calcinosis can be present. Arthritis and mechanic’s hands may occur but are less pronounced than in antisynthetase syndrome (25).

Internal organ risk — ILD is common, often NSIP. Compared with ATA-ILD, PM-Scl–ILD tends to follow a more indolent course with better long-term lung outcomes and survival under standard care. Cardiac involvement is uncommon but reported in overlap cases (25). SRC is rare.

Clinical course — Myositis activity can wax and wane with immunomodulatory therapy; lung disease generally stabilizes or responds. Long-term outcomes are typically favorable compared with ATA-ILD when monitored and treated systematically.

### Ku

Clinical phenotype — Overlap phenotype with myositis features (proximal weakness, myalgia) and non-erosive inflammatory arthritis (26, 27). Raynaud phenomenon is common; skin fibrosis can be limited or absent (28). Mechanic’s hands may be seen.

Internal organ risk — ILD is frequent (often NSIP) (26), ranging from subclinical to progressive. Myocardial inflammation is reported rarely. PAH risk is not specifically elevated beyond overlap context. GI dysmotility follows general SSc patterns.

Clinical course — Many patients respond to immunomodulatory treatment of muscle and lung disease, though heterogeneity is substantial. Regular PFT/HRCT and CK assessments are recommended.

### RuvBL1/2

Clinical phenotype — Uncommon but appears SSc-specific. Often associates with dcSSc and myositis overlap (proximal weakness; myopathic enzymes) (29).

Internal organ risk — ILD can occur but is less well quantified; cardiac involvement is not well defined. No specific renal or malignancy signals are established.

Clinical course — Data are limited; follow-up should prioritize muscle and lung monitoring. Extended panels can reclassify seronegative patients with compatible phenotypes.

### U1 RNP

Clinical phenotype — Characteristic of mixed connective tissue disease (MCTD) yet found in SSc, indicating overlap features. Puffy fingers, Raynaud phenomenon, synovitis, and myositis are common; esophageal dysmotility and reflux occur frequently. Skin fibrosis may be limited or sine in overlap presentations.

Internal organ risk — Pulmonary arterial hypertension is reported more frequently than in some other serotypes; ILD varies from minimal to moderate (30). Myocarditis is uncommon but not absent. SRC is rare.

Clinical course — The trajectory reflects overlap biology: variable flares of arthritis/myositis and gradual cardiopulmonary involvement. Regular PAH screening (echo) and ILD assessment (PFT/HRCT as indicated) are prudent.

### U11/U12 RNP

Clinical phenotype — Anti−U11/U12 (anti−RNPC−3) antibodies are uncommon (~3%) but SSc−specific; patients often present with early cough/dyspnea and variable skin involvement (31).

Internal organ risk — Strongly associated with ILD that is frequently severe/progressive, with early FVC/DLCO decline (32). Possible GI dysmotility and malignancy signals need confirmation.

Clinical course — Prioritize early, intensive ILD surveillance (baseline HRCT, frequent PFTs) and consider early referral to an ILD−expert center. Confirmatory testing (IP) is advised for weak line−blot reactivity (31, 32).

### Emerging or novel SSc-specific autoantibodies

Anti-NVL antibody has recently been identified as a novel disease-specific autoantibody in systemic sclerosis. It typically exhibits a nucleolar pattern on indirect immunofluorescence using HEp-2 cells and has been detected in both Japanese and Western cohorts. Interestingly, its clinical associations appear to differ between ethnic groups, further emphasizing the importance of considering population background when interpreting serological findings in SSc (33–36).

Some other novel and rare autoantibodies have been reported in systemic sclerosis, such as anti-eIF2B (37–39) and anti-BICD2 (40). These antibodies are currently limited to research-level assays and are not yet incorporated into routine diagnostic testing. Their prevalence and clinical significance remain to be established, and at present, they should be regarded as emerging serological markers rather than clinically validated tools.

### Regional perspectives: Japan

RNAP III and SRC — Anti–RNAP III positivity markedly increases SRC risk in Japanese cohorts; higher ELISA indices and IP subsets further stratify risk (13). Seronegative SSc (41–43) — A Japanese single-center series found ≈10% ANA/SSc-autoantibody–negative patients with distinct features (44). Early-onset severe SSc — A multicenter prospective cohort identified predictors of clinical features in early-onset severe SSc (45).

### Regional and ethnic cohort differences: Asian vs. Europe/North America

The distribution of SSc-specific autoantibodies shows regional variation even within Asia (46).

Japanese cohorts report a relatively high prevalence of ACA, while anti–RNAP III antibodies are consistently rare compared with Western cohorts (47). In contrast, large Chinese cohorts have demonstrated very high frequencies of ATA (up to ~60%) and anti–U1RNP (~18%), with low frequencies of ACA (~13%) and RNAP III (~2%) (48). These patients often present with diffuse cutaneous SSc and a high burden of interstitial lung disease (ILD). Studies from Korea also show ATA predominance, with ATA-positive patients exhibiting higher risks of diffuse skin involvement and pulmonary complications (49) A multicenter Hong Kong cohort reported ILD prevalence close to 50%, with crackles and elevated CRP as independent predictors of ILD development and progression. Long-term follow-up (median 8 years) revealed a mortality rate of ~24%, underscoring the prognostic burden of lung involvement (50). Data from Southeast Asia further support this trend. In a Thai cohort, high levels of anti–topoisomerase I were associated with a shorter interval from Raynaud’s onset to cardiopulmonary involvement, underscoring the aggressive lung and heart disease trajectory in ATA-positive patients (6). ACA was infrequent and RNAP III nearly absent, aligning with broader Asian patterns.

Thus, Asian cohorts (including Japan) often show lower RNAP III prevalence and higher ATA/U1 RNP proportions, contributing to a greater ILD burden and smaller RNAP III–defined subset, when compared with European/North American cohorts (51). Despite lower prevalence, the effect sizes for SRC and cancer clustering among RNAP III–positive patients are comparable cross-region (13–15, 17, 52, 53). ACA–PAH associations are consistent worldwide (4, 54). U3 RNP associates with African ancestry and is relatively rare in Asia (55, 56).

Taken together, these data highlight both diversity and convergence within Asia. While frequencies differ across countries, several shared features emerge: (i) ATA predominance, (ii) lower prevalence of ACA and RNAP III, and (iii) a consistently high burden of ILD. These findings collectively outline an “emerging Asian clinical trend” Such regional comparisons emphasize that genetic background, environmental exposures, and local testing practices jointly shape the observed serological landscape. In contrast, European cohorts have consistently demonstrated higher frequencies of ACA and RNAP III antibodies. For example, the South Australian Scleroderma Register (52) and multicenter cohorts from Italy and France (51, 53) report ACA prevalence exceeding 30–40% and RNAP III rates of 10–20%, markedly higher than in most Asian cohorts. These data reinforce the geographic divergence: ACA and RNAP III are more common in Europe, whereas ATA predominates in Asia.

Since methodological differences (IP vs LIA/ELISA/CBA) can mimic geographic biology, assay platform and cut-off alignment are essential for fair comparisons.

### Assay performance and practical testing strategy

Line immunoassay (LIA) offers multi-antigen throughput but shows antigen-specific variability versus IP or single-antigen ELISAs; weak positives in low pre-test settings account for many false positives. It is recommended: (1) use LIA when SSc probability is at least moderate (57); (2) confirm Th/To, U3 RNP/fibrillarin, PM-Scl, Ku, and borderline ATA with orthogonal assays; and (3) for RNAP III, add quantitative ELISA and, where available, IP subset assignment.

### Toward standardized autoantibody measurement: a global proposal

Clinical-first standardization — Two components are already broadly standardized: (i) ANA by IIF under ICAP nomenclature; and (ii) a core ELISA panel (ACA, ATA/Scl-70, RNAP III, U1 RNP). These enable comparable first-line results across regions and laboratories (**Table 1**).

**Table 1:** SSc-related autoantibodies—organ involvement and clinical course, monitoring, and confirmatory testing.

Antibody	IIF ANA pattern (ICAP) (66, 67)	Typical subset	Major organ risks	Monitoring focus	Confirmatory testing
ACA (CENP-A/B)	Centromere (discrete) — AC-3 (66)	lcSSc ≫ dcSSc	PAH, digital ischemia, calcinosis; ILD relatively lower	Echo-based annual PAH pathway; baseline HRCT+PFT	If atypical: ELISA; consider IP when discordant
ATA (Scl-70)	Nuclear speckled — AC-29 (66, 68)	dcSSc ≫ lcSSc	ILD onset/progression; cardiac involvement	HRCT baseline; PFT q3–6mo; early therapy when progressing	Borderline LIA→ ELISA/IP
RNAP III	Coarse speckled; nucleolar uncommon — AC-10/AC-4/5 (66)	dcSSc > lcSSc	SRC, rapid skin, malignancy near onset	Home BP; early ACE-I; minimize GC; cancer vigilance	Quantitative ELISA; IP subset (I/II/III)
U3 RNP (Fibrillarin)	Nucleolar (clumpy) — AC-9 (18, 66)	dcSSc, nucleolar	PAH/ILD; vasculopathy; multi-organ	Right heart & lung surveillance	CBA/ELISA; IP confirmation
Th/To	Nucleolar (homogeneous/punctate) — AC-8 (69)	lcSSc	ILD/PAH (center-dependent)	ILD/PAH surveillance	IP recommended when LIA weak
NOR90 (hUBF)	Nucleolar (punctate ‘NOR’ dots) — AC-10 (70)	lcSSc	Inconsistent organ signals (ILD)	Symptom-driven	ELISA ± IP as needed
PM-Scl (75/100)	Nucleolar (granular) — AC-8 (66, 71)	Overlap	ILD & myositis; often favorable course	ILD & muscle monitoring	ELISA/IP esp. if LIA weak
Ku	Fine speckled — AC-4/5 (66)	Overlap	ILD & myositis	ILD & muscle monitoring	Confirm discordant LIA with IP/ELISA
RuvBL1/2	Typically nuclear speckled (72)	dcSSc/overlap	Myositis overlap	Phenotype-driven	Extended panels
U1 RNP	Nuclear coarse speckled — AC-5 (31)	Overlap/MCTD-like	PAH; ILD variable; GI dysmotility	Echo-based PAH screening; ILD as indicated	ELISA/IP as needed
U11/U12 RNP	Nuclear speckled (varied) — AC-2/4/5 (31)	varied	Severe ILD; GI dysmotility; cancer (some reports)	Intensified ILD surveillance	Specialized assay/IP

*ICAP ANA pattern definitions:* AC-3 (Centromere): discrete centromere speckles in interphase nuclei and metaphase centromeres (66, 67). AC-4 (Fine speckled): numerous fine speckles in interphase nuclei; AC-5 (Coarse speckled): larger/coarser speckles. AC-8 (Homogeneous nucleolar): smooth nucleolar staining; AC-9 (Clumpy nucleolar): coarse/clumpy nucleoli; AC-10 (Punctate nucleolar, NOR pattern): dots over nucleolar organizer regions (66, 67).

Pattern-guided reflexing — If ANA is positive, ICAP patterns inform pre-test probabilities (centromere → ACA; nucleolar → U3 RNP/Th/To/NOR90; speckled → U1 RNP/others). Based on the clinical picture, proceed to reflex confirmation with line immunoassay (line-blot) or immunoprecipitation (IP). Recently, a novel multiplex protein array–based platform (Autoantigen-Capture-Coupled Bead Array “A-Cube”) has been developed for comprehensive serological profiling of SSc autoantibodies (58). Although still in an experimental phase, this technology may complement conventional line-blot or immunoprecipitation assays by enabling simultaneous, quantitative analysis of multiple specificities, and could contribute to future standardization of reflex testing.

Harmonization priorities — While ANA and core ELISAs are relatively standardized, cross-platform harmonization is most needed for the reflex layer: (a) per-antigen calibrators and shared reference sera for LIA/IP; (b) phenotype-anchored cut-offs with an ‘equivocal’ (gray) zone; (c) transparent per-antigen performance reporting; (d) external QA participation; and (e) published reflex-testing algorithms linked to risk-based monitoring bundles.

### Pragmatic algorithm (Text)

**Figure 1:**
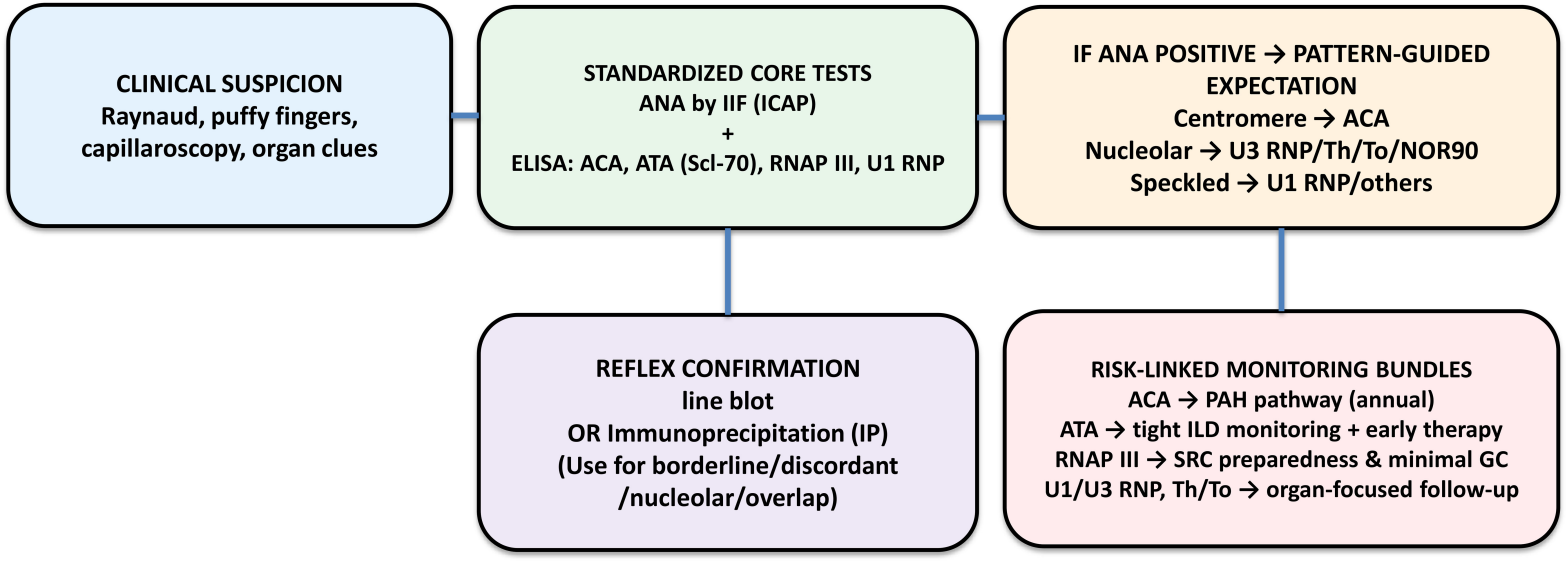
Clinical-first standardized workflow (ANA/ICAP + core ELISAs → line blot/IP confirmation). ICAP, International Consensus on ANA Patterns; LIA, line immunoassay; ELISA, enzyme-linked immunosorbent assay; IP, immunoprecipitation; PAH, pulmonary arterial hypertension; ILD, interstitial lung disease; SRC, scleroderma renal crisis.

1) Clinical suspicion → ANA (ICAP) + core ELISAs (ACA, ATA, RNAP III, U1 RNP). 2) If ANA positive, use ICAP pattern to refine pre-test probabilities. 3) Reflex confirmation with LIA or IP when results are borderline/discordant or for nucleolar/overlap targets (Th/To, U3 RNP, PM-Scl, Ku, Nor90) (57). 4) Link antibody profiles to monitoring bundles: ACA→PAH; ATA→ILD (59); RNAP III→SRC (13); U1/U3/Th/To→organ-focused follow-up (**Figure 1**).

### Pathogenic and therapeutic implications

The intrinsic pathogenicity of systemic sclerosis–specific autoantibodies remains controversial, and many aspects of their involvement in disease mechanisms are still unclear (60). Experimental data suggest that anti–topoisomerase I antibodies can stimulate fibroblasts and promote extracellular matrix deposition, which may explain their strong clinical association with progressive interstitial lung disease (61, 62). Anti–RNA polymerase III antibodies, in contrast, are temporally linked with cancer occurrence and scleroderma renal crisis, although direct mechanistic evidence is limited (63).

At the same time, autoantibody measurement has become firmly established in clinical practice for diagnostic classification and for guiding investigations. While correlations such as ATA titers with disease severity support a contributory role, decisive proof of direct pathogenicity and therapeutic targeting remains lacking (64). Recent advances in B-cell–directed approaches, particularly those targeting CD19 or CD20, have shown clinical promise in severe systemic sclerosis. These strategies do not directly neutralize circulating autoantibodies but instead act by selectively eliminating the antibody-producing cells—akin to dismantling the factories of autoantibody production through immune effector mechanisms (65).

Whether such interventions ultimately confirm a direct pathogenic role for SSc-specific autoantibodies or reveal them primarily as bystanders of immune dysregulation remains unresolved. This tension between biomarker and driver continues to represent one of the central immunological impacts in systemic sclerosis, and it is likely to guide future investigation.

## Conclusions

While their direct pathogenic role remains debated, SSc-specific autoantibodies continue to serve as the clinical foundation (60). A clinical-first standardized workflow—anchoring on ANA (ICAP) and core ELISAs with pattern-guided reflex LIA/IP—can harmonize measurement across regions and sharpen risk-adapted management.
